# The effects of short‐term caloric restriction on cardiometabolic health in overweight/obese men and women: A single‐arm trial

**DOI:** 10.14814/phy2.15856

**Published:** 2023-11-20

**Authors:** Justin A. DeBlauw, Anna I. Churchill, Brigitte C. Yunda, Christopher J. Kotarsky, Abigail Caldwell, Stephen J. Ives

**Affiliations:** ^1^ Health and Human Physiological Sciences Skidmore College Saratoga Springs New York USA

**Keywords:** adipokines, adiposity, fasting, fat oxidation, hormones, metabolism, weight loss

## Abstract

Overweight and obesity (Ow/Ob) is a risk factor for cardiometabolic disease. Caloric restriction (CR) have been investigated but little is known about the acute effects of CR and often such diets are not standardized. Thus, we aimed to assess the impact of a new standardized 3‐day CR diet (590 kcal/d intake) on cardiometabolic health in weight‐stable Ow/Ob individuals. In a single‐arm design, 15 Ow/Ob men and women were assessed pre‐post a 3‐day standardized CR diet; specifically, body weight/composition (%body fat, visceral fat score (Vfs), blood pressure (BP), and vascular stiffness (VS), resting energy expenditure (REE), substrate utilization (respiratory quotient, RQ), and blood glucose/lipid profile). CR lowered body weight (93.1 ± 15.2 to 90.67 ± 14.4 kg, *p* < 0.001, *d* = 1.9), %fat (37.2 ± 7.5 to 35.8 ± 7.5%, *p* = 0.002, *d* = 1.1), and Vfs (13.1 ± 4.5 to 12.2 ± 3.9 a.u., *p* = 0.002, *d* = 1.1), but not body water (46.3 ± 3.6 to 46.0 ± 3.6%, *p* = 0.29). CR lowered VS (29.8 ± 17.5 to 21.5 ± 14.5%, *p* = 0.05, *d* = 0.6), but not BP (*p* > 0.05). Blood glucose (86 ± 7 to 84 ± 11 mg/dL, *p* = 0.33) and lipids (total cholesterol (196 ± 49 to 203 ± 54 mg/dL, *p* = 0.16) and TC/HDL (4.9 ± 2.4 to 6.1 ± 4.7, *p* = 0.13)) were unchanged. RQ decreased with CR (0.84 ± 0.01 to 0.76 ± 0.00, *p* < 0.001, *d* = 1.9), though REE was unchanged (*p* = 0.83). The 3‐day CR diet significantly improved fat metabolism, body weight and composition, and vascular stiffness.

## 1 INTRODUCTION

According to the Centers for Disease Control (CDC), more than 42% of adults are classified as obese, and approximately 74% of US adults are classified as overweight (body mass index of 25.0 to <30 kg/m^2^) or obese (>30 kg/m^2^) (Ow/Ob) (Fryar et al., 2020). The costs on human health are profound, increasing the risk of heart disease, stroke, Type 2 diabetes, and certain cancers, and ultimately increasing morbidity associated with these chronic diseases. Obesity and the negative sequalae inflict staggering economic costs, estimated at $261 billion, and that was in 2016 (Cawley et al., 2021). At the same time, the weight loss supplement market, is estimated $66 billion (Grebow, 2021), most of which are not assessed for efficacy. Therefore, understanding commercially available dietary interventions may help address Ow/Ob, the risk of chronic disease, and health care costs.

Obesity is associated with physiological disruption, specifically metabolic dysfunction such as impaired ability to use fat or metabolic inflexibility (Kelley et al., 1999). Interventions, beyond alteration of diet, typically involve exercise. However, prior systematic review and meta‐analysis suggests that when comparing diet alone versus exercise alone, dietary interventions result in approximately threefold greater weight loss (11 vs. 3 Δkg) (Miller et al., 1997). While the additional benefits of exercise are not be discounted (Giallauria et al., 2021), engaging dietary approaches can be a highly effective way to promote weight loss. Caloric restriction (CR) has long been touted as an effective method of inducing weight loss (Walford et al., 2002), though the degree of restriction can vary from ca. 25% reduction in caloric intake to a very low‐calorie diet (VLCD) of 400–800 kcal/d absolute intake (Saris, 2001). Caloric restriction, not a new concept or experience for humankind, the early studies from the Dutch famine during World War II (Elias et al., 2005; Strøm et al., 1951), cultural comparisons of the United States and Okinawa, Japan (Everitt & Le Couteur, 2007), and the Biosphere 2 study (Walford et al., 2002) have documented that CR improves cardiometabolic risk profile (e.g., blood pressure, lipids, etc.). The prominent Comprehensive Assessment of Long‐term Effects of Reducing Calorie Intake (CALERIE) trial, is a multicenter longitudinal controlled trial of CR (Kraus et al., 2019; Most et al., 2017; Ravussin et al., 2015). CR of 20–25% reduced body weight and adiposity, lowered blood pressure, and improved lipid profile in “non‐obese” healthy individuals (body mass index 22–27.9 m^2^) (Kraus et al., 2019). Though long‐term sustained CR may induce health improvements, supported by study of CR society members (Fontana et al., 2004, 2010), it does not present without potential physiological issues (e.g., risk of osteoporosis, reproductive dysfunction, etc.; Dirks & Leeuwenburgh, 2006) and concerns about adherence and maintenance (Wadden, 1993). Thus, more investigation into possible benefit of short‐term CR is warranted.

More recent work on short‐term (1–2 weeks) CR (700–1000 kcal/d intake) in Ow or Ob women suggests improved body weight and body composition CR (Jakobsdottir et al., 2016; Kautzky et al., 2021). Though the simple act of reducing caloric intake over time with CR, or intermittent fasting (IF), results in caloric deficit and contributes to weight loss in Ow/Ob (Harris et al., 2018; Most & Redman, 2020). Such caloric deficit engages the “metabolic switch” (Patikorn et al., 2021), which is the subsequent transformation of liver metabolism, “switching” from predominately carbohydrate/glucose in the liver/muscle to ketones derived from adipocytes, and further adaptations such as improved ability to cope with metabolic stress and glucose handling (Miller et al., 1997). The time course for the metabolic switch has been suggested to be 72 h, but studies of short‐term CR often do not assess changes in substrate utilization (Jakobsdottir et al., 2016; Kautzky et al., 2021). Given the impaired ability of Ow/Ob to oxidize fat (Kelley et al., 1999), metabolic switching could improve the ability to utilize fat (Grajower & Horne, 2019), and might reduce fat mass. CR benefits extend beyond weight loss (Most & Redman, 2020), also likely influencing hormonal regulators of metabolism (PYY, NPY (Holzer et al., 2012), thyroid hormones, ghrelin, and leptin (Klok et al., 2007)), which are known to be disrupted in Ow/Ob (Lean & Malkova, 2016). To date, no studies have tested 3 days of CR (<600 kcal/day intake) using a standardized diet in Ow/Ob individuals, as might be included in a long‐term IF diet, to see it improves weight/adiposity, metabolic switching (Goodpaster & Sparks, 2017), and potential alterations in the hormonal regulators of appetite and metabolism (e.g., protein YY (PYY), neuropeptide Y (NPY), etc.).

Accordingly, the purpose of this single‐arm trial was to document the effects of a 3‐day very low‐calorie CR diet (<600 kcal/d) on body weight/composition, measures of cardiometabolic health as well as circulating regulatory factors that regulate hunger, satiety, and metabolism. We hypothesized that the 3‐day CR diet would induce favorable changes in body weight, body composition, metabolic function, via increased fat use, blood pressure, and blood glucose/lipid profile. This study could provide evidence on the acute efficacy of a novel 3‐day CR on body weight/composition, but also identify the potential underlying physiological mechanisms through which such dietary intervention may elicit cardiometabolic improvement. Collectively, this may provide a preliminary data for study of this diet with IF.

## 2 METHODS

### 2.1 Subjects and general procedures

**FIGURE 1:**
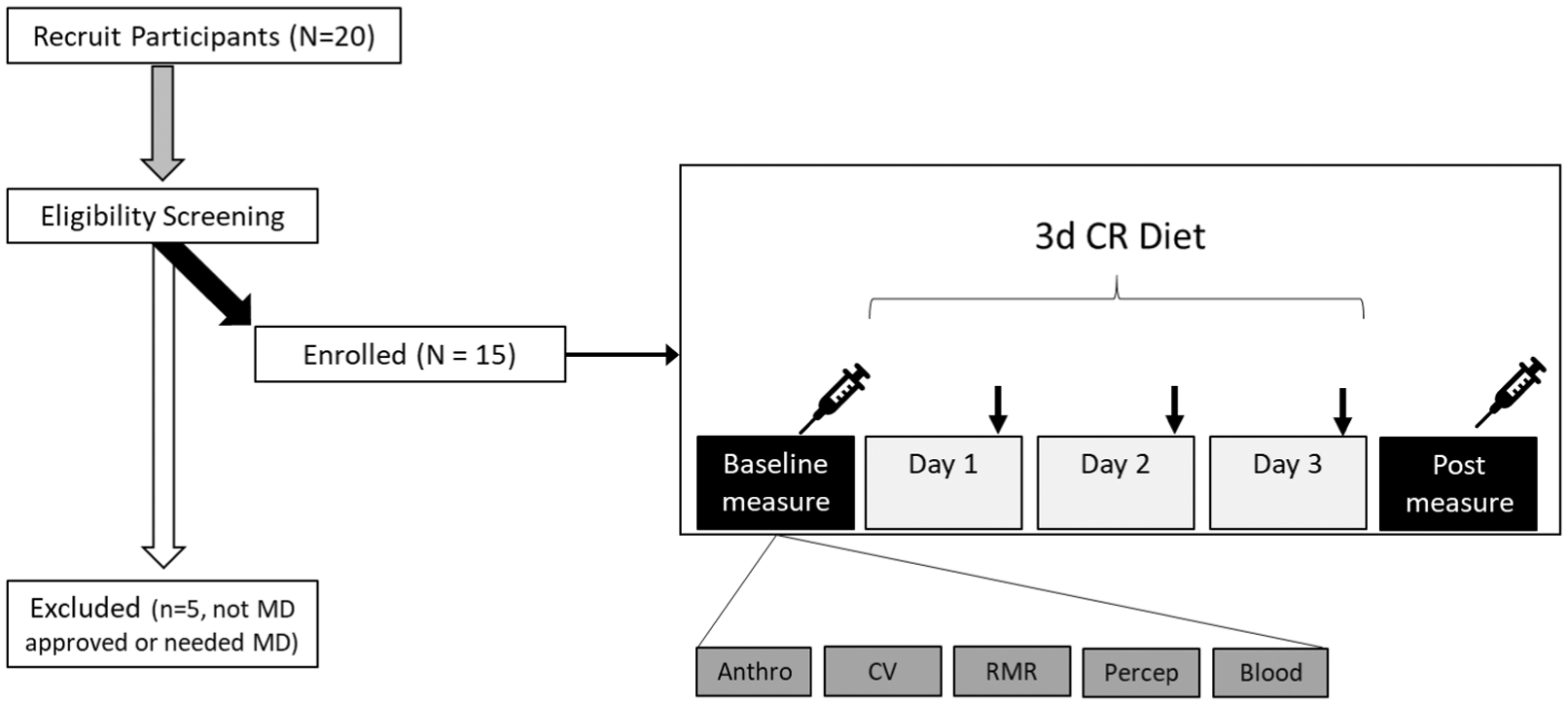
Experimental overview. ↓ perceptual survey was completed.

Participants were recruited via email and publicly posted flyers from the Saratoga Springs, NY community. To be included participants must have been Ow or Ob (defined as BMI > 27.5 kg/m^2^), and weight stable (±2 kg) for ≥6 months prior, and 25–65 years of age. Participants were otherwise relatively healthy without uncontrolled chronic disease (e.g., cardiovascular, metabolic, or pulmonary) and two or fewer positive risk factors for cardiovascular disease (e.g., high blood pressure, high cholesterol, etc.) as described by the American College of Sports Medicine/American Heart Association Criteria (Pescatello et al., 2021). Participants were screened for eligibility by health history form in person prior to baseline measurements (Figure 1). Additionally, considering the use of CR, we sought clearance from participants' physicians regarding an individual's suitability for participation. Thus, to be included participants also must have been cleared by their physician. Subjects with more than two CVD risk factors or have uncontrolled/overt cardiovascular, pulmonary, or metabolic disease (diabetes mellitus), recent blood donation (<8 weeks), who have cancer or are being treated for cancer, or history of an eating disorder, or food allergies were excluded. To ensure greater ecological validity and representation, women were included in this study; however, we did not control for menstrual cycle phase, as done previously (Dudar et al., 2020). Women who were currently pregnant, breastfeeding, attempting to conceive, or amenorrheic (not associated with menopause) were excluded from the study given the use of CR. Subjects were asked to continue any currently prescribed medications, but avoid any supplements (e.g., vitamins, nutraceuticals [herbs, extracts, etc.], weight loss pills, etc.) prior to and during the study. Participants were also asked to avoid strenuous exertion, and/or alcohol intake throughout the duration of the study, and to avoid strenuous exercise for 24 h and caffeine/alcohol intake for 12 h preceding the pre‐post visits. Participants provided written informed consent prior to participation. This study was reviewed and approved by the Institutional Review Board at Skidmore College (#2204‐1028) and registered with clinicaltrials.gov (NCT05422391).

### 2.2 Procedures

This study was an open‐label, single‐arm study design, see overview of the study procedures in Figure 1. Once deemed eligible and cleared by their physician, participants were then scheduled to come into the lab for baseline testing, prior to starting the diet. Participants came into the Human Nutrition and Metabolism laboratory at Skidmore College between 06:30 and 07:30 a.m. (Monday or Tuesday), under standardized conditions described above, and were asked to arrive hydrated with water only. Upon arrival, height, weight, waist/hip circumference (waist at level of umbilicus/hip widest portion of buttocks; Brown et al., 2018), and total body composition (fat and lean mass, body water) were assessed using a stadiometer (Seca, Mt. Pleasant, SC, USA), Gulick tape measure (Gulick II Plus), and body composition scale (RD‐545, Tanita, Arlington Heights, IL, USA) (Vasold et al., 2019), respectively. The Tanita BIA is known to be reliable and valid approach (Vasold et al., 2019), and in‐house testing yielded an average coefficient of variation of 0.4% across three trials. The average, in‐house testing, coefficient of variation for waist and hip circumferences was 0.54%. Participant BMI was confirmed as meeting eligibility criteria prior to any further testing, and all subjects met the criteria.

Participants were then positioned supine, and allowed to rest for 10 min, during this time we instrumented them with an oscillometric blood pressure cuff. Peripheral blood pressure, estimated central blood pressure and pulse wave analysis (Augmentation Index, AIx) (Shoji et al., 2017) were assessed using oscillometric cuff technique, in duplicate and then averaged. To determine the potential influence of the 3‐day CR on metabolism, we measured resting metabolic rate (volume of oxygen consumed, VO_2_) using indirect calorimetry and ventilated hood technique (Haugen et al., 2003) with a metabolic cart (TrueOne2400, Parvomedics, Sandy, UT, USA) (Crouter et al., 2006). From the metabolic cart data we then estimated relative substrate utilization (%fat and % carbohydrate) (Frayn, 1983). After completing metabolic testing, participants completed visual analog scales (0–100 mm line) to assess perceptions of hunger, satiety, fullness, and desire to eat (Flint et al., 2000), using online survey software (QualtricsXM, Boston, MA, USA). These perceptual measures were repeated nightly each day over the study and to assess compliance participants were also asked to report what time consumed each of diet as part of this survey. Participants were asked to complete the consensus sleep diary (CSD) core form (Carney et al., 2012), again on the Qualtrics online platform.

Finally, participants were transported to the Health Services department to have a blood sample taken from an upper extremity. A small aliquot (40 μL) of fresh blood was used for blood lipid and glucose measurement using the cholestech ldx analyzer (Abbott, Lake Forest, IL, USA), which has been validated against standard clinical testing (Carey et al., 2006). In brief, total cholesterol, high‐density lipoprotein (HDL), low‐density lipoprotein (LDL), HDL/total cholesterol ratio, triglycerides, and glucose were assessed. The average coefficient of variation for the lipid panel during in‐house testing was 1.9%. Remaining blood in the EDTA tubes was centrifuged and plasma aliquoted for storage at −80°C for later analysis of biomarkers.

Participants were then given the standardized diet, which was the Plexus 3‐day Reset diet program (Plexus Worldwide LLC, Scottsdale, AZ), and this marked the start of Day 1 (Monday or Tuesday). Details of the diet are provided in Tables S1 and S2. Participants followed the diet on this day and 2 subsequent days, and then came in on the fourth day (Thursday or Friday) to repeat the same set of tests as baseline (“post‐diet”). The very low‐calorie diet itself approximates 590 kcal/day (when prepared as directed), is low fat (<30% of Kcal from fat), and is taken with 68–84 ounces of fluid (ca. 10 cups or 2.5 L of fluid, mostly from water). The diet also provides three “meals” or boluses of protein in the recommended 20–40 g range to sustain muscle protein synthesis (Kerksick et al., 2017), but is also associated with improvements in body weight/composition (Arciero et al., 2014). Additional clear liquids were allowed ad libitum. As a monitor of adherence to the diet, the nightly online survey asked if/when they consumed the various contents of the diet. Participants were instructed to not alter their physical activity patterns, but to avoid exercise.

### 2.3 Biomarker analysis

To estimate in vivo biological effects of the 3‐day CR, plasma samples were analyzed using commercially available assay kits for ketone bodies (b‐hydroxybutyric acid, BOH; and acetoacetic acid, AcAc; Sigma Aldrich, Burlington, MA), factors that regulate metabolism (insulin, thyroid stimulating hormone, cortisol; RayBiotech, Peachtree, GA), and factors that influence hunger/satiety (leptin, ghrelin, protein YY, neuropeptide Y; RayBiotech, Peachtree, GA). From glucose, mentioned above, and insulin the homeostatic model of assessment of insulin resistance (HOMA‐IR) was calculated using published formula (Matthews et al., 1985). All assays were completed in duplicate with standard curve linearity *r* ^2^ > 0.9, and coefficient of variation (cv) of <8%, and any values outside of the standard curve were not included in the final analysis.

### 2.4 Data and statistical analysis

In a paired samples *t*‐test model, using a one‐tail approach, *α* = 0.05, large effect size (Cohen's *d* = 0.8), to yield an acceptable power of 0.8, an estimated 12 subjects would be needed (G*Power, Dusseldorf, Germany). We assumed that by recruiting 20, this could allow for possible dropout/non‐compliance ensuring beyond the minimally effective sample size. Data were analyzed using open source software (JASP v.16.4.0, Amsterdam, Netherlands). The data were analyzed using paired samples t‐tests and a complementary estimate of effect size, Cohen's d, where 0.2, 0.5, and ≥0.8 represent small, medium, and large effect sizes, respectively. Perceptual data over time were analyzed using a one‐way repeated measures analysis of variance. Assumptions for these tests were run and if a violation to normality occurred, a nonparametric alternative was employed, or an adjustment to the degrees of freedom was made. Alpha was set to 0.05. Data are presented as means ± standard deviation, unless otherwise noted.

## 3 RESULTS

### 3.1 Participants

The fifteen individuals who completed the study were nearly middle aged, with a relatively even split of men and women (Table 1). According to the body mass index (BMI), they indeed met the criteria of being classified as Ow or Ob, but appeared otherwise healthy. Using the CR‐diet protein intake (77 g) and the average body weight, the relative protein intake was 0.82 g/kg of body weight/day which meets the recommended daily allowance (RDA). Though men and women were both included in this study, aside from the expected differences at baseline in height/weight, exploratory statistical analysis suggested no difference in how men and women responded to the 3‐day CR diet, thus the data are combined. Compliance to the diet, as assessed by self‐reported questionnaire was 100%.

**TABLE 1:** Participant characteristics (*n* = 15).

Characteristic	Mean ± SD
Age (years)	47 ± 9
Sex (male/female)	7/8
Height (cm)	168.7 ± 10.3
Weight (kg)	93.6 ± 15.1
Body mass index (kg/m^2^)	33.2 ± 4.3
Waist‐hip ratio	0.9 ± 0.1
Body fat (%)	37.5 ± 7.3
Systolic BP (mm Hg)	118 ± 8
Diastolic BP (mm Hg)	73 ± 5

### 3.2 Impact of 3‐day CR diet on body weight, body composition, and waist/hip circumferences

**FIGURE 2:**
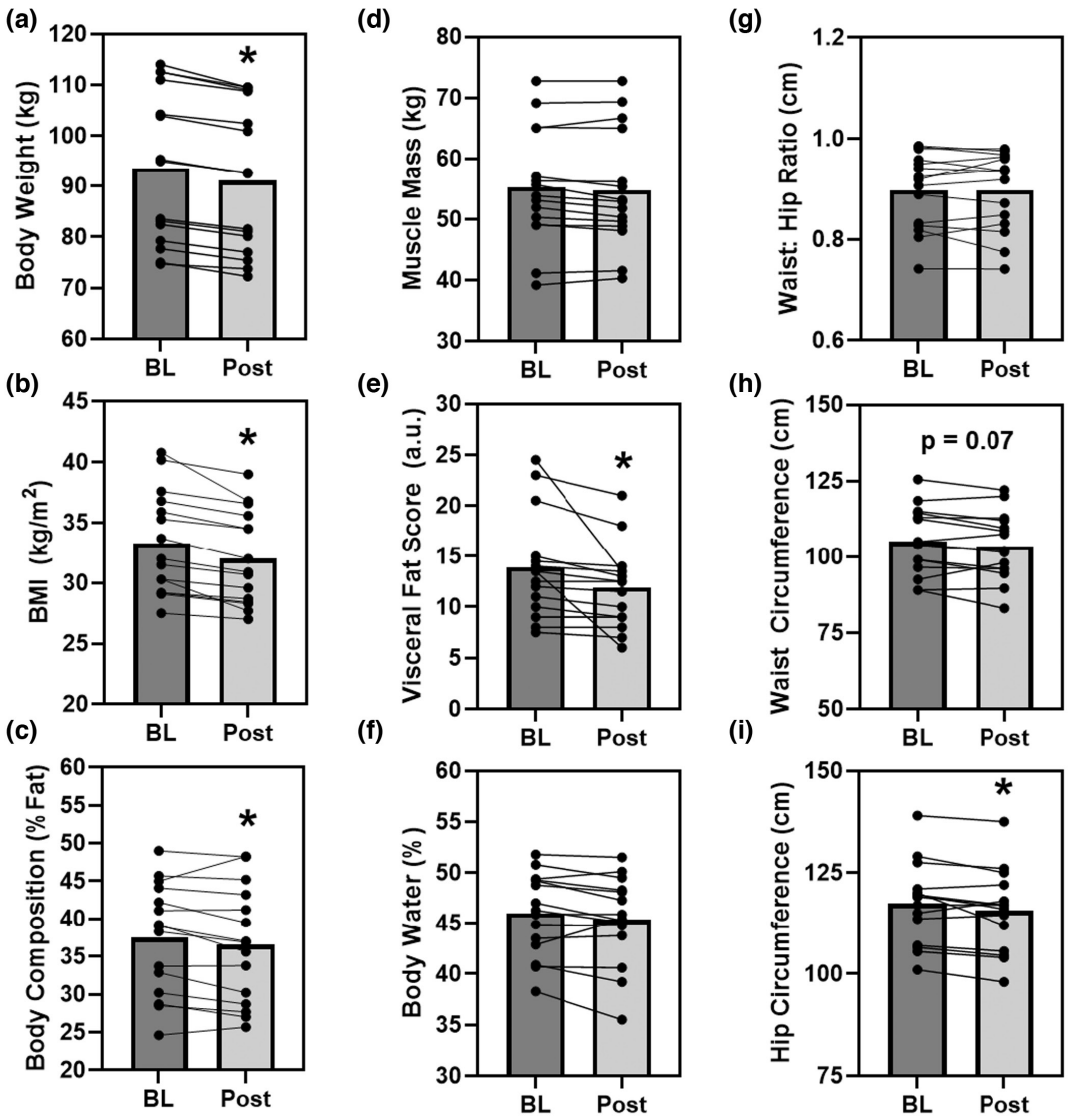
The effect of the 3‐day CR diet on body weight (Panel a), body mass index (BMI, Panel b), body composition (Panels c–f), and waist‐hip circumferences (Panel g–i) in overweight/obese men and women (*n* = 15). *p < 0.05 pre versus post. Lines are individuals, bars are means.

The 3‐day CR diet significantly lowered body weight by 3% (*p* < 0.001, *d* = 1.9), body mass index by 3% (*p* < 0.001, *d* = 1.4), body fat (*p* = 0.002, *d* = 1.1), and visceral fat score by 14% (*p* = 0.002, *d* = 1.1) (Figure 2). Waist (*p* = 0.07, *d* = 0.5) and hip (*p* = 0.02, *d* = 0.7) circumferences were lower as well, by ca. 1% each, thus waist‐hip ratio was unaffected (*p* = 1.0, *d* = 0, Figure 2). No significant changes were observed in muscle mass (*p* = 0.13, *d* = 0.4) or body water (*p* = 0.29, *d* = 0.3) in response to the 3‐day CR diet (Figure 2).

### 3.3 Impact of 3‐day CR diet on blood pressure and pulse wave analysis

**FIGURE 3:**
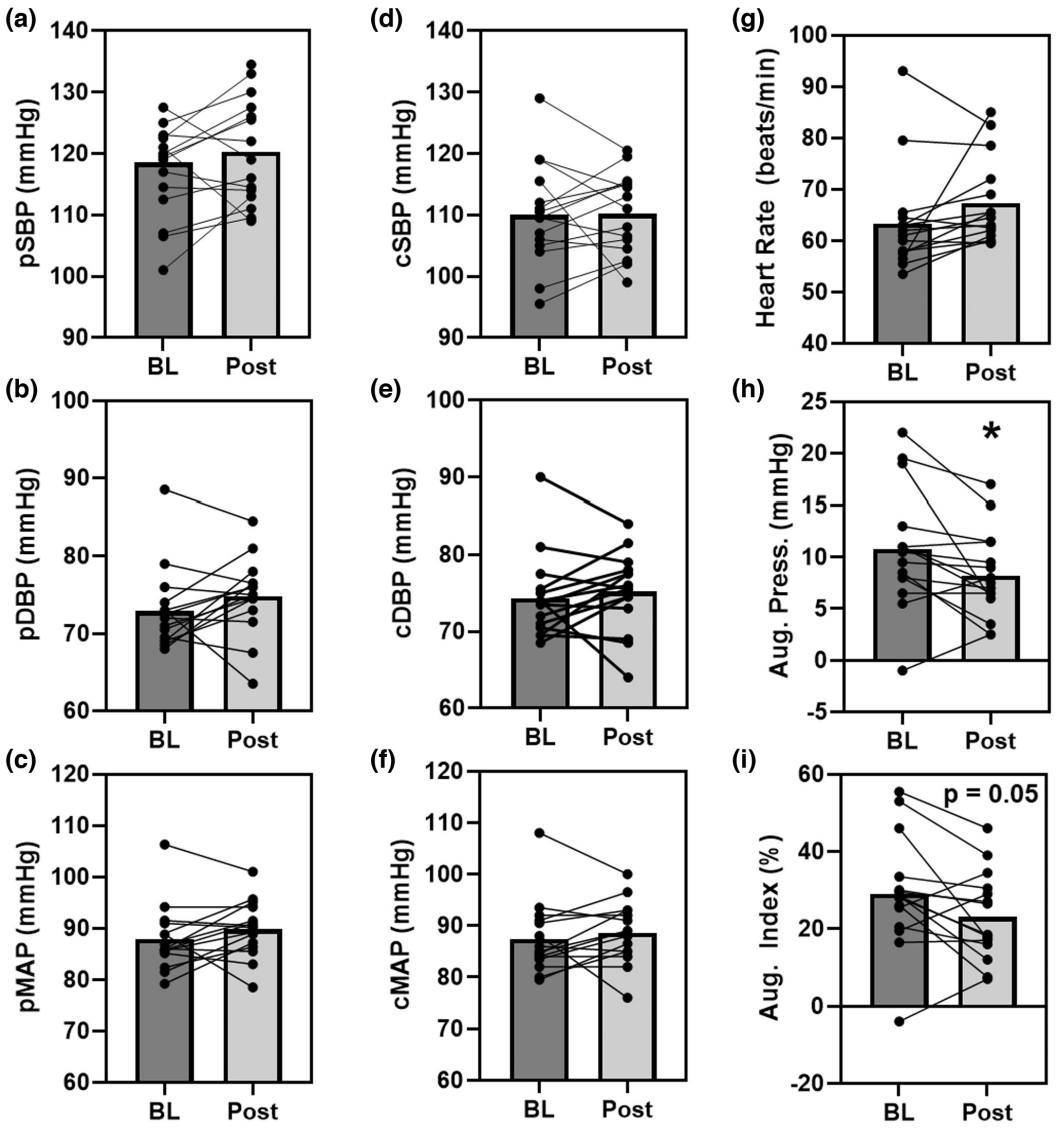
The effect of the 3‐day CR diet on peripheral (p, Panel a–c) and central (c, Panel d–f) systolic (SBP), diastolic (DBP), and mean arterial (MAP) blood pressures, heart rate (Panel g), augmentation pressure (Panel h), and augmentation index (Panel i) in overweight/obese men and women (*n* = 15). *p < 0.05 pre versus post. Lines are individuals, bars are means.

The 3‐day CR diet had no significant effect on peripheral systolic (pSBP; *p* = 0.36, *d* = 0.2), diastolic (pDBP; *p* = 0.16, *d* = 0.4), or mean (pMAP; *p* = 0.23, *d* = 0.3) blood pressures (Figure 3, left panel). This was also true for estimated central systolic (cSBP; *p* = 0.94, *d* = 0.0), diastolic (cDBP; *p* = 0.43, *d* = 0.2), or mean (cMAP; *p* = 0.41, *d* = 0.2) blood pressures (Figure 3, central panel). Heart rate was not significantly different with the 3‐day CR diet (HR; *p* = 0.08, *d* = 0.2). The pulse wave analysis revealed that while the 3‐day CR diet had no effect on pulse pressure (*p* = 0.56, *d* = 0.1), it did significantly alter augmentation pressure (AP; *p* = 0.03, *d* = 0.6) and augmentation index (29.8 ± 17.5 to 21.5 ± 14.5%, *p* = 0.05, *d* = 0.6) (Figure 3, right panel).

### 3.4 Impact of 3‐day CR diet on blood lipids and glucose

**FIGURE 4:**
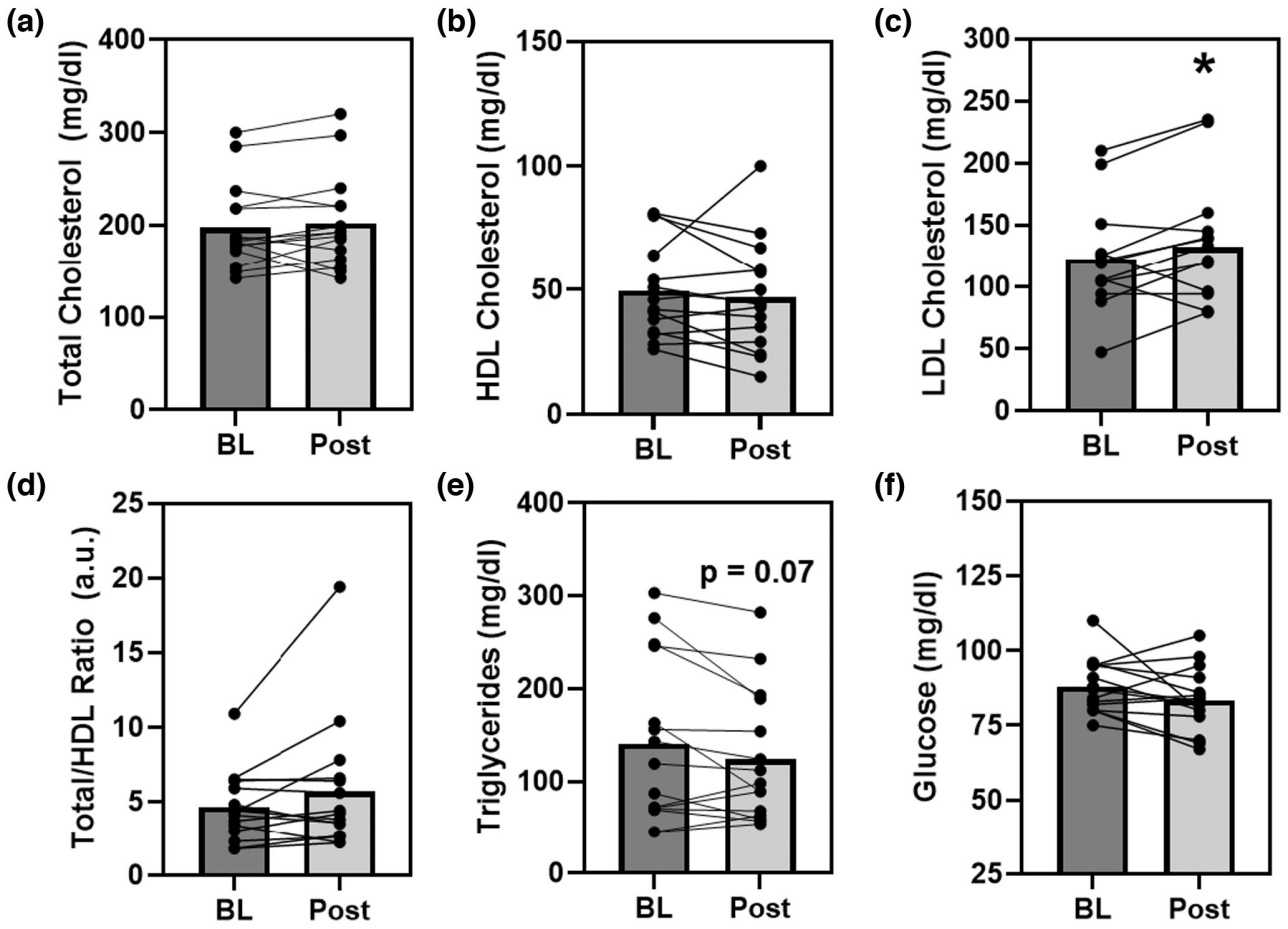
The effect of the 3‐day CR diet on blood lipids (Panels a–e) and glucose (Panel f) in overweight/obese men and women (*n* = 15). *p < 0.05, pre versus post. Lines are individuals, bars are means.

The 3‐day CR diet had no significant effect on blood glucose (*p* = 0.11, *d* = 0.4), total cholesterol (*p* = 0.37, *d* = 0.2), HDL cholesterol (*p* = 0.42, *d* = 0.2), or TC/HDL (*p* = 0.14, *d* = 0.4) (Figure 4). Although triglycerides tended to decrease (*p* = 0.07, *d* = 0.5) and LDL cholesterol tended to increase (*p* = 0.05, *d* = 0.6) (Figure 4) in response to the 3‐day CR diet in Ow/Ob men and women.

### 3.5 Impact of 3‐day CR diet on metabolism and relative substrate utilization

**FIGURE 5:**
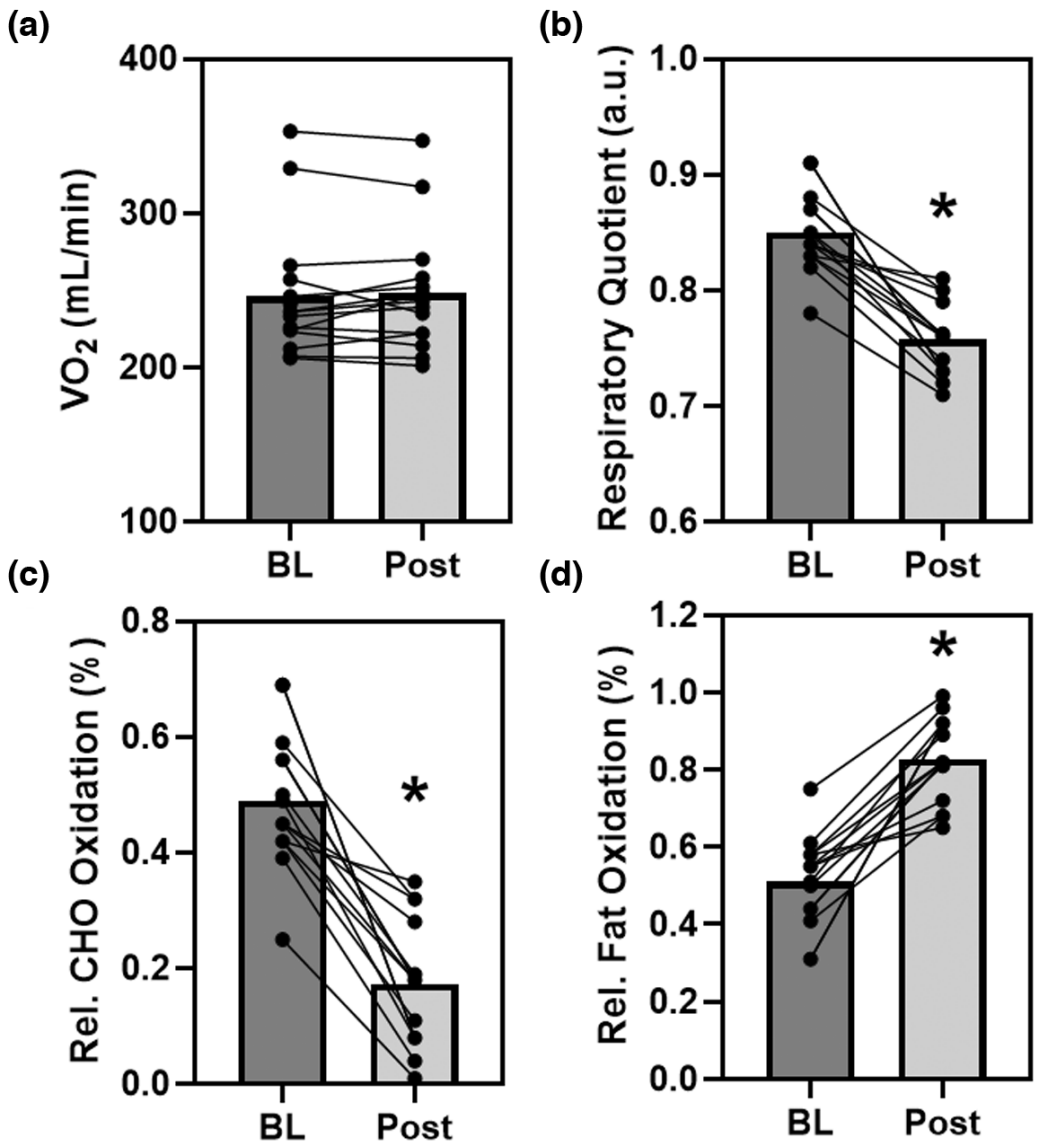
The effect of the 3‐day CR Diet on resting metabolism (volume of oxygen consumed, VO_2_, Panel a), respiratory quotient (RQ, Panel b), and relative substrate oxidation (carbohydrate, CHO; and fat, Panel c, d) in overweight/obese men and women (*n* = 15). *p < 0.05 pre versus post. Lines are individuals, bars are means.

The 3‐day CR diet had no significant effect on VO_2_ (*p* = 0.28, *d* = 0.3, Figure 5) and thus resting energy expenditure (1718 ± 274 to 1722 ± 317 kcal/d, *p* = 0.83, *d* = 0.1), which is derived from the VO_2_. Though a significant reduction in the respiratory quotient (RQ) was observed (*p* < 0.001, *d* = 2.1) suggesting metabolic shift from using carbohydrates to fats (Figure 5). Indeed, further quantification of relative substrate utilization indicated an ca. 65% reduction in the relative use of carbohydrates (*p* < 0.001, *d* = 2.1) and corresponding 38% increase in the amount of fat oxidation (*p* < 0.001, *d* = 2.1) (Figure 5).

### 3.6 Impact of 3‐day CR diet on circulating metabolic factors

The 3‐day CR diet had no significant effect on 3‐hydroxybutryic acid, cortisol, insulin, neuropeptide Y, ghrelin, protein YY, or on sirtuin 1 (all: *p* > 0.05, *d* < 0.4, Table 2). The ketone acetoacetic acid (*p* = 0.04, *d* = 1.0) and leptin (*p* = 0.02, *d* = 0.6) were significantly increased in response to the 3‐day CR diet in Ow/Ob men and women (Table 2). Using the glucose and insulin parameters, HOMA‐IR was calculated although was not significantly changed following the 3‐day CR diet (*p* = 0.49, *d* = 0.2, Table 2).

**TABLE 2:** Blood assay biomarker analysis.

Parameter (units)	Time	Concentration	*p*‐value	Effect size
Acetoacetic acid (mM)	Pre	0.7 ± 0.3	—	—
	Post	1.0 ± 0.2	0.04	1.0
3‐hydroxybutryric acid (mM)	Pre	2.0 ± 1.3	—	—
	Post	2.2 ± 1.3	0.54	0.2
Cortisol (ng/mL)	Pre	15.0 ± 4.8	—	—
	Post	15.2 ± 4.0	0.32	0.0
Insulin (μU/mL)	Pre	19.0 ± 16.6	—	—
	Post	16.4 ± 13.8	0.58	0.2
Leptin (pg/mL)	Pre	158 ± 8	—	—
	Post	230 ± 121*	0.02	0.6
NPY (ng/mL)	Pre	16.6 ± 6.8	—	—
	Post	15.5 ± 7.1	0.23	0.3
Ghrelin (pg/mL)	Pre	749 ± 37	—	—
	Post	728 ± 68	0.49	0.3
Protein YY (ng/mL)	Pre	0.33 ± 0.35	—	—
	Post	0.36 ± 0.59	0.61	0.1
SIRT1 (ng/mL)	Pre	70.9 ± 42	—	—
	Post	63.9 ± 39	0.33	0.4

*Note*: Data are mean ± SD. *p<0.05 pre vs. post. Effect size is Cohen's d (≤0.2 small or trivial, 0.5 moderate, ≥0.8 large effect).

### 3.7 Impact of 3‐day CR diet on perceptions of hunger, fullness, and self‐reported sleep

The 3‐day CR diet had no significant effect on self‐reported perceptions or feelings of hunger, desire to eat, or fullness (all: *p* > 0.05, data not shown). Satiety did vary over time and seemed to reach a high point at the end of Day 2, which then decreased at Day 3, and to post‐diet testing on Day 4 (data not shown). The 3‐day CR diet had no significant effect on self‐reported sleep latency, or the total sleep duration over the course of the study (both *p* > 0.05, data not shown).

## 4 DISCUSSION

In the current study we aimed to see how an acute 3‐day very low‐calorie CR diet might affect body weight/ composition, metabolism, cardiovascular health, and circulating metabolic factors which may explain changes in these parameters in Ow and Ob men and women. Three days of CR resulted in significant weight loss, loss of total body fat/visceral fat, and loss in waist and hip circumference, without significant changes in body water or muscle mass. These anthropometric changes might be supported by switching from reliance on carbohydrates to oxidizing more fat as fuel, which could explain, along with increased circulating leptin, the loss of fat mass. There were minimal changes in blood pressure and blood lipids/glucose, suggesting nominal effects, although augmentation pressure and index, indicators of aortic stiffness responded favorably to the intervention. In terms of individual perceptions of the effects of short‐term CR on hunger or fullness, there was not much effect, other than satiety which peaked on Day 2 of the diet. Self‐reported sleep latency and duration were also unaffected. Collectively, the findings from the current study suggest that 3 days of CR acutely induces weight and fat loss, increases circulating leptin and resting fat oxidation, without much change to cardiovascular health, feelings of hunger, or sleep in adult men and women who are Ow or Ob. Considering these findings, such a short‐term very low‐calorie CR diet could be an effective way to induce the onset of weight loss, or be incorporated as part of a long‐term intermittent fasting diet approach to weight loss, but further studies are warranted to determine efficacy in an IF model.

### 4.1 Impact of short‐term CR on body weight and composition

Caloric restriction is not a novel approach of losing weight, in those that desire to do so, and has even been purported to induce health benefits beyond weight loss (Fontana et al., 2010; Holloszy & Fontana, 2007; Jakobsdottir et al., 2016; Kautzky et al., 2021; Kraus et al., 2019; Ravussin et al., 2015). The CALERIE trial might be one of the more prominent controlled trials of long CR, achieving 12% reduction in caloric intake, yielded an average weight loss 7.5 kg over 2 years in healthy weight men and women (Kraus et al., 2019). In Ow and Ob, a review of CR (15–60% reduction in intake) indicated that daily CR resulted in reductions of body weight and fat mass loss were 5–8%, and 10–20%, respectively (Varady, 2011). However, less is known about the temporal nature of these responses. One study that explored the acute and chronic impact of caloric restriction (ca. 1000 kcal/d) in obese women, observed significant reductions in waist circumference and tendency for lower fat mass in the first week, though body weight was not reported (Jakobsdottir et al., 2016). This prior study examined the temporal response using 1‐week epochs and only measured women. A more recent study, again in women, of CR (700 kcal intake) over 2 weeks observed significant reductions in BMI, %body fat, and psychological parameters, though the study included multiple interventional parameters (e.g., counseling, massage, etc.) (Kautzky et al., 2021). In the current study of Ow/Ob men and women, using 3 days of more aggressive CR or very low‐calorie diet (VLCD, ca. 600 kcal), we observed a 3% reduction in body weight, 3% reduction in %body fat, a 14% reduction in visceral fat score, and 1% reduction in waist/hip circumferences (Figure 2). Thus, VLCD CR may induce benefits earlier on than might be expected. Interestingly, muscle mass and body water were not significantly impacted by the 3‐day CR. This is perhaps due to the protein pacing (multiple protein intakes over the day) in the range of 20–40 g/meal that is recommended to sustain muscle protein synthesis (Kerksick et al., 2017), which has been shown to be effective in weight loss and lean mass maintenance (Arciero et al., 2014; Arciero, Edmonds, et al., 2016; Arciero, Ives, et al., 2016). Further, prescribed fluid intake with this standardized diet and is taken with 68–84 ounces of fluid (ca. 10 cups or 2.5 L of fluid, mostly from water), with possible additional ad libitum fluid intake, which may explain the lack of significant change in body water. These aspects of the diet, protein pacing, and sufficient fluid intake, may be able to sustain body water and lean mass, at least over this 3‐day duration.

This 3‐day CR diet may be a way to commence an individual's weight loss in a distinct but effective way, but perhaps more importantly could be part of an intermittent CR or intermittent fasting dietary approach to weight loss and body composition improvement. Consumers without knowledge or training may struggle to identify adequate dietary approaches to weight loss, such as IF, and the dietary planning associated with them, therefore an “off the self” product or suite of products could make adopting such a weight loss strategy easier. Thus, findings from the current study may provide insight into a new paradigm of IF (4:3, 4 days ad libitum, 3 days caloric restriction using the 3‐day diet) and provide evidence on an “off the shelf” way of carrying out a version of IF, as many individuals may struggle to plan and effectively carry out such dietary interventions. Such an IF approach might avoid complications associated with chronic/continuous CR (Dirks & Leeuwenburgh, 2006), especially very low‐calorie diets over the long term, which may be inadvisable. This low‐fat diet which maintains minimally recommended protein intake, ensures adequate intake of fluids and electrolytes, and leverages various supplements, such as fiber and other plant‐derived factors (e.g., green coffee beans) could be beneficial for those that are looking to lose weight. Future studies should explore this in larger, and more diverse, groups to confirm these findings, and how long such body weight and composition changes may last with this acute 3‐day bout of CR.

### 4.2 Impact of short‐term CR on cardiovascular health

Long‐term caloric restriction has been demonstrated to induce favorable cardiovascular effects, namely reductions in blood pressure and improved lipid profile (Fontana et al., 2004; Holloszy & Fontana, 2007; Jakobsdottir et al., 2016; Nicoll & Henein, 2018; Walford et al., 2002), even in nonobese (Ravussin et al., 2015), though intermittent fasting (IF) may elicit similar effects (Arciero et al., 2022; Yang et al., 2021). Long‐term CR of ca. 25% in magnitude lowered systolic and diastolic blood pressure, decreased total and LDL cholesterol, lowered triglycerides, lowered total cholesterol/ HDL cholesterol ratio, and increased HDL in “non‐obese” healthy individuals (BMI of 22–27.9 m^2^) (Kraus et al., 2019). Although these reductions with modest CR (Kraus et al., 2019), and those reported with IF (Yang et al., 2021), are minimal, on the order of a couple mm Hg reduction in BP or fractions of 1 mmol/L cholesterol or triglyceride, only with more extreme CR are more dramatic reductions in these parameters observed. For example, in humans upward of 40% reduction in caloric intake, over many years, can reduce systolic and diastolic BP by ca. 20 mm Hg and improve lipid profile by >30% in normal weight individuals (Fontana et al., 2004). Focusing on those who are Ow or Ob, and a more acute perspective, on a 1000 kcal/d diet, obese women saw reductions in systolic BP of ‐10 mm Hg, diastolic BP of −4 mm Hg in a 4‐week time span, with only fractional changes in triglycerides (Jakobsdottir et al., 2016). Interestingly, systolic BP was significantly reduced by ca. 6 mm Hg with a week of CR onset, suggesting that CR may induce rapid changes in systolic BP.

Although, in the present study, using a short‐term 3‐day CR down to 590 kcal/d of intake we observed no significant changes in systolic, diastolic, or mean arterial blood pressure measured peripherally or estimated centrally at the aorta (Figure 3). This extension of prior work, examining more acute response to CR, and using oscillometric cuff technique and generalized transfer function to estimate central pressures and pulse wave analysis provides novel insight. Especially considering that central pressures are independent predictors of cardiovascular events and mortality (Vlachopoulos et al., 2010), and provide more detailed investigation into possible effects on the heart better than peripheral BP (Kollias et al., 2016). Interestingly, the 3‐day CR lowered augmentation pressure and index (Figure 3), suggestive of a cardioprotective or beneficial effect (Vlachopoulos et al., 2010), although prior investigations have found that longer bouts of CR had no such effect on augmentation index or other parameters of vascular stiffness (Petersen et al., 2016; Weiss et al., 2016). Thus, this reduction in augmentation pressure and index may be beneficial but also might be an acute phase response that may normalize over time or may be specific to the macro‐ and micronutrient content of the diet employed in CR or initial weight status of the study population.

In terms of lipid profile, while longer term CR may induce reductions in total cholesterol, LDL cholesterol, triglycerides, and glucose with concomitant increases in HDL cholesterol, thereby improving the total/HDL ratio (Jakobsdottir et al., 2016; Walford et al., 2002; Yang et al., 2021) the effects with low‐intensity CR are modest. In the current study, we observed no significant changes in total, HDL, total/HDL ratio, or in blood glucose, but we did observe a significant increase in LDL cholesterol (Figure 4). Although prior studies have indicated that acute fasting or CR may increase LDL, which may be related to weight loss (Akaberi et al., 2014; Sävendahl & Underwood, 1999). Examination of insulin and glucose independently and together in the HOMA‐IR (Table 2) indicated no effect of the 3‐day CR on insulin sensitivity and glucose homeostasis. Further analysis of the blood for cardiovascular health relevant parameters, namely the CR‐sensitive but vasoprotective sirtuin‐1 (SIRT1) (Csiszar et al., 2009), revealed no significant effects of the 3‐day CR diet on SIRT1 levels (Table 2). Thus, the 3‐day CR diet had a minimal or perhaps predictable effect on parameters germane to cardiovascular health (e.g., blood pressure and lipids/glucose), but tended to reduce estimates of central arterial stiffness in Ow/Ob men and women.

### 4.3 Impact of short‐term CR on metabolism and substrate utilization

Obesity is associated with impaired ability to use fat or metabolic inflexibility (Kelley et al., 1999). CR and/or IF elicits favorable adaptations through the concept of the “metabolic switch” where altered caloric intake subsequently transforms liver metabolism “switching” from predominately carbohydrate/glucose in the liver/muscle to free fatty acids and ketones derived from adipocytes (Anton et al., 2018). Further adaptations with CR may be observed, such as improved ability to cope with cellular or metabolic stress and glucose handling (Patikorn et al., 2021), but CR‐induced “metabolic switching” may increase ability to utilize fat (Grajower & Horne, 2019). The metabolic switch timing occurs in the 12–36 h timeframe after commencing a fast, depending on time to deplete glycogen. Indeed, in the present study of Ow/Ob men and women we found that at baseline fasting or basal fat oxidation was relatively lower than carbohydrates (Figure 5), an effect which was reversed following the 3‐day CR diet. This shift in substrate utilization via increased fat oxidation, or metabolic switching occurred independent of any change in resting metabolic rate as assessed by VO_2_. It is perhaps this increased fat oxidation, via metabolic switch, which contributed to the aforementioned weight loss and more specifically fat loss observed in the present study. Mechanistically, analysis of blood samples revealed no significant effect of the 3‐day CR diet on circulating levels of insulin, 3‐hydroxybutyric acid, cortisol, neuropeptide Y, protein YY, or ghrelin (Table 2). However, we did observe significant increases in acetoacetic acid (a ketone) and circulating levels of leptin, although variable changes in leptin with fasting have been reported (Abdullah et al., 2020; Jakobsdottir et al., 2016), such increases may be, in part, responsible for the greater lipolysis and fat oxidation (Harris, 2014). While a tenable hypothesis, the current data and underlying measurements are limited to whole body estimates of substrate metabolism and future more invasive studies are required to confirm.

### 4.4 Impact of short‐term CR on perceptions of hunger, satiety, and sleep

There is less known about the effects of acute or short‐term CR on perceptions hunger/desire to eat or satiety/fullness, although longer term CR is known to induce psychological predilection to hunger and less so to feelings of satiety or fullness (Anton et al., 2009). In the present study we found no significant effects of the short‐term 3‐day CR diet on feelings of hunger, desire to eat, or fullness; although, satiety seemed change peaking on the second day of the diet. Although the appetite and hunger‐related hormones NPY, PYY, and ghrelin were all unchanged with the 3‐day CR diet in Ow/Ob, the elevation in leptin, though temporally or kinetically misaligned, may be related to this self‐reported increase in satiety (Most & Redman, 2020). While perhaps unrelated to hunger/fullness, we sought to determine the potential effects of short‐term CR on sleep, and using self‐reported sleep parameters, latency and duration, we observed no significant effects of the 3‐day CR diet on sleep in Ow/Ob men and women.

### 4.5 Limitations

The present study, as with every study, was not conducted without limitations. As a secondary or tertiary outcome, in assessing sleep we opted for a less invasive option to minimize participant burden, which does relate significantly to sleep duration, even if perhaps imperfectly (Dietch & Taylor, 2021). During the intervention participants were released on their own recognizance and expected to comply with the diet, and its suggested timing, and the self‐reported survey data suggest near 100% compliance to the diet, which is supported by the fact that all participants lost weight. Participant pre‐study diet and physical activity levels were not controlled for or measured. While these are limitations, this approach is more ecologically valid than direct inpatient or clinical center monitoring. The sample size is limited, and cannot represent the exact expected response among a larger and more heterogenous population. The ad hoc sample size estimation indicated 12 to achieve adequate power, with 15 having completed the study, coupled with reporting of actual p values and effect sizes, reduces concern over sample size, although further work is warranted. The single‐arm trial design, while using participants as their own control (pre‐intervention weight stable ad libitum dietary status vs. post‐intervention status) capitalizes on a within subjects design, using a randomized controlled trial is advisable for future studies of this 3‐day CR diet. Measuring beyond the post period is also necessary to determine if the CR‐induced changes are sustained. Women were included in this study and menstrual status (other than requiring a normal period, for premenopausal only) was not controlled for, though ecologically valid, it is a limitation of the study.

## 5 CONCLUSION

In summary, the findings from the current study highlight that 3 days of a novel standardized very low‐calorie diet CR (ca. 590 kcal/d intake) induces significant weight and fat loss, increases circulating leptin, increases fat oxidation, without much change to blood lipid profile, blood pressure, feelings of hunger, or sleep in adult men and women who are Ow or Ob. These novel findings suggest that such a diet could be beneficial as an approach to initiating weight loss in those that are Ow or Ob, or could be utilized in an intermittent fasting diet (e.g., alternate day or 5:2) approach to weight loss, but further studies are warranted to determine efficacy and whether such weight loss is sustained.

## AUTHOR CONTRIBUTIONS

Conceptualization, JD and SI; methodology, JD and SI.; formal analysis, AC, BY, JD, SI.; investigation, JD, AC, BY, CK, AC, SI.; resources, SI AC; data curation, AC, BY, JD, SI.; writing—original draft preparation, JD, SI, CK; writing—review and editing, JD, SI, AC, BY CK AC.; visualization, AC BY SI; supervision, SI JD; project administration, SI.; funding acquisition, SI. All authors have read and agreed to the published version of the manuscript.

## FUNDING INFORMATION

This research was funded by Plexus Worldwide, grant number 2204‐1028.

## DISCLOSURES

Plexus Worldwide provided funding and product for the current study although the funders had no role in the design of the study; in the collection, analyses, or interpretation of data; in the writing of the manuscript; or in the decision to publish the results.

## ETHICS STATEMENT

All participants provided written informed consent prior to participation. This study was reviewed and approved by the Institutional Review Board at Skidmore College (#2204‐1028) and registered with clinicaltrials.gov (NCT05422391). This study was carried out in accordance with the principles set forth in the most recent revisions to the Declaration of Helsinki.

## Supporting information


Table S1.

Table S2.

## Data Availability

The data are available upon reasonable request by the corresponding author.
