# Supplementary material for: The effects of short‐term caloric restriction on cardiometabolic health in overweight/obese men and women: A single‐arm trial
Source: Physiol Rep. 2023 Nov 20;11(22):e15856. doi: 10.14814/phy2.15856 (PMC10659943; doi:10.14814/phy2.15856)
Supplement: Supplementary file 1 — Table S1. [file PHY2-11-e15856-s002.docx]

| Table S1. Macronutrient composition of 3-d CR Diet | | | |  |  |  |
| --- | --- | --- | --- | --- | --- | --- |
| Item | Energy (Kcal) | Fat (g) | Protein (g) | CHO (g) | Total Sugars (g) | Dietary Fiber (g) |
| Morning drink | 10 | 0 | 0 | 1 | 0 | 0 |
| Breakfast shake | 230 | 3.5 | 30 | 24 | 15 | 5 |
| Mid-morning drink | 15 | 0 | 0 | 8 | 0 | 6 |
| Lunch Soup | 80 | 0 | 20 | 1 | 0 | 0 |
| Snack bar | 100 | 4 | 7 | 18 | 1 | 5 |
| Afternoon drink | 25 | 0 | 0 | 6 | 3 | 0 |
| Dinner soup | 110 | 0 | 20 | 8 | 1 | 1 |
| Evening drink | 20 | 0 | 0 | 4 | 0 | 0 |
| **TOTAL** | **590** | **7.5** | **77** | **70** | **20** | **17** |
