# Supplementary material for: The effects of short‐term caloric restriction on cardiometabolic health in overweight/obese men and women: A single‐arm trial
Source: Physiol Rep. 2023 Nov 20;11(22):e15856. doi: 10.14814/phy2.15856 (PMC10659943; doi:10.14814/phy2.15856)
Supplement: Supplementary file 2 — Table S2. [file PHY2-11-e15856-s001.docx]

| Table S2. Micronutrient composition of 3-d CR Diet | | | | |  | |  | |  | |  | |  | |  | |  | |
| --- | --- | --- | --- | --- | --- | --- | --- | --- | --- | --- | --- | --- | --- | --- | --- | --- | --- | --- |
| Item | Morning drink | Breakfast shake | Mid-morning drink | Lunch Soup | | Snack bar | | Afternoon drink | | Dinner soup | | Evening drink | | **TOTAL** | | **%RDA** | |  |
| Vitamin C (mg) | 170 | 13.5 | 0 | 0 | | 54 | | 0 | | 0 | | 0 | | 237.5 | | 263 | |  |
| Vitamin D (mcg) | 0 | 3.7 | 0 | 0 | | 0 | | 0 | | 0 | | 0 | | 3.7 | | 19 | |  |
| Vitamin A (mcg) | 0 | 247 | 0 | 0 | | 270 | | 0 | | 0 | | 0 | | 517 | | 57 | |  |
| Vitamin E (mg) | 0 | 2.5 | 0 | 0 | | 21 | | 0 | | 0 | | 0 | | 23.5 | | 157 | |  |
| Vitamin B6 (mg) | 0 | 0.4 | 0 | 0 | | 1.7 | | 0 | | 0 | | 0 | | 2.1 | | 123 | |  |
| Vitamin B12 (mcg) | 0 | 1.7 | 0 | 0 | | 4.8 | | 0 | | 0 | | 0 | | 6.5 | | 271 | |  |
| Calcium (mg) | 70 | 480 | 0 | 74 | | 0 | | 0 | | 0 | | 0 | | 624 | | 48 | |  |
| Iron (mg) | 0 | 4.2 | 0 | 1 | | 0 | | 0 | | 0 | | 0 | | 5.2 | | 29 | |  |
| Potassium (mg) | 200 | 715 | 0 | 110 | | 0 | | 0 | | 0 | | 35 | | 1,060 | | 23 | |  |
| Magnesium (mg) | 50 | 88 | 0 | 0 | | 0 | | 0 | | 0 | | 0 | | 138 | | 33 | |  |
| Zinc (mg) | 3 | 3.3 | 0 | 0 | | 0 | | 0 | | 0 | | 0 | | 6.3 | | 57 | |  |
| Chloride (mg) | 70 | 0 | 0 | 0 | | 0 | | 0 | | 0 | | 0 | | 70 | | 3 | |  |
| Thiamin (mg) | 0 | 0.4 | 0 | 0 | | 1.5 | | 0 | | 0 | | 0 | | 1.9 | | 158 | |  |
| Riboflavin (mg) | 0 | 0.7 | 0 | 0 | | 2.8 | | 0 | | 0 | | 0 | | 3.5 | | 269 | |  |
| Niacin (mg) | 0 | 7.4 | 0 | 0 | | 20 | | 0 | | 0 | | 0 | | 27.4 | | 169 | |  |
| Folate (mcg) | 0 | 96 | 0 | 0 | | 0 | | 0 | | 0 | | 0 | | 96 | | 24 | |  |
| Pantothenic Acid (mg) | 0 | 0.35 | 0 | 0 | | 10 | | 0 | | 0 | | 0 | | 10.35 | | 207 | |  |
| Phosphorus (mg) | 0 | 370 | 0 | 0 | | 0 | | 0 | | 0 | | 0 | | 370 | | 30 | |  |
| Iodine (mcg) | 0 | 134 | 0 | 0 | | 0 | | 0 | | 0 | | 0 | | 134 | | 89 | |  |
| Selenium (mcg) | 0 | 23 | 0 | 0 | | 0 | | 0 | | 0 | | 0 | | 23 | | 42 | |  |
| Molybdenum (mcg) | 0 | 35 | 0 | 0 | | 0 | | 0 | | 0 | | 0 | | 35 | | 78 | |  |
| Choline (mg) | 0 | 45 | 0 | 0 | | 0 | | 0 | | 0 | | 0 | | 45 | | 8 | |  |
| Polydextrose (mg) | 0 | 0 | 6,250 | 0 | | 0 | | 0 | | 0 | | 0 | | 6,250 | | -- | |  |
| Sodium (mg) | 0 | 0 | 0 | 0 | | 5 | | 0 | | 0 | | 0 | | 5 | | <1 | |  |
| Manganese (mg) | 0 | 1 | 0 | 0 | | 0 | | 0 | | 0 | | 0 | | 1 | | 43 | |  |
| Chromium (mcg) | 0 | 0 | 200 | 0 | | 0 | | 0 | | 0 | | 0 | | 200 | | 571 | |  |
